# Migration of Vapor Molecules in Soils

**DOI:** 10.3390/molecules31183245

**Published:** 2026-09-14

**Authors:** Olga Kudryashova, Vladimir Gruznov, Andrey Kikhtenko, Alexander Vorozhtsov

**Affiliations:** 1 Faculty of Physics and Engineering, Tomsk State University, 634050 Tomsk, Russia; abv1953@mail.ru; 2 Trofimuk Institute of Petroleum Geology and Geophysics of Siberian Branch Russian Academy of Sciences, 630090 Novosibirsk, Russia; gruznovvm@ipgg.sbras.ru (V.G.); kikhtenkoav@ipgg.sbras.ru (A.K.)

**Keywords:** molecular transport, vapor diffusion, unsaturated porous media, soil moisture, sorption, effective diffusion, pore structure, low-volatility organic compounds, environmental monitoring

## Abstract

Molecular transport of low-volatility organic vapors through unsaturated porous media is governed by the coupled effects of diffusion, sorption, and pore structure, yet the relative roles of these processes remain insufficiently quantified. In this work, we develop a physics-based model describing vapor migration from a subsurface source to the soil surface by explicitly accounting for moisture-dependent sorption, air-filled porosity, and pore clogging by fine particles. The model predicts that soil moisture affects vapor transport through two competing mechanisms: thin water films progressively suppress gas–solid sorption, thereby increasing the effective diffusion coefficient, whereas further wetting reduces the connectivity of air-filled pores and ultimately blocks gas-phase transport. As a consequence, vapor migration exhibits a non-monotonic dependence on soil moisture, with a distinct optimum for surface vapor flux. The model further predicts that fine particles substantially decrease vapor transport by reducing pore connectivity, while lower temperatures suppress migration through both reduced molecular diffusivity and enhanced sorption. Laboratory experiments using representative low-volatility energetic compounds through sand with controlled moisture content and particle composition confirmed all major qualitative predictions of the model, including enhanced transport at intermediate moisture, suppression under dry, dusty and highly saturated conditions, and strong temperature dependence. Although energetic compounds were used as representative low-volatility substances, the proposed framework is generally applicable to molecular transport of trace organic vapors in unsaturated porous media and provides a quantitative basis for predicting environmental conditions under which subsurface sources can be detected by surface vapor measurements.

## 1. Introduction

The transport of volatile and semi-volatile molecules through porous media plays a central role in many natural and technological processes, including soil–atmosphere exchange, environmental monitoring, pollutant migration, subsurface gas emissions, and geochemical exploration [1]. To predict whether trace amounts of vapor emanating from a subsurface source can reach the ground surface, it is necessary to understand the combined effects of molecular diffusion, gas–solid sorption, soil moisture, and pore structure.

The migration of semi-volatile vapors through porous media has its own unique characteristics. In practical terms, studying the transport processes of semi-volatile molecules is of particular importance in the mining industry during storage, transportation, and drilling and blasting operations. The release of volatile components from buried sources results in the formation of a chemical signature on the soil surface, which can be used in environmental monitoring of contaminated areas [2].

For low-volatile compounds, molecular transport, rather than vaporization, is often the limiting process determining surface concentration. In the presence of a subsurface source, vapor traces may remain undetected because transport is strongly influenced by environmental conditions. The ability of molecules from a subsurface source to reach the soil surface is primarily determined by competition between transport and retention within the porous medium.

When studying vapor transport through soil, high-energy compounds are often chosen as model analogs due to their extremely low vapor pressure. A wide range of analytical methods has been proposed for detecting trace amounts of such compounds. The most common are ion mobility spectrometry (IMS), gas chromatography with an electron capture detector (GC-ECD), gas chromatography–mass spectrometry (GC-MS), and fluorescent polymer sensors [2,3,4,5,6,7,8,9]. Modern sensor systems are capable of detecting concentrations of nitroaromatic compounds at the ppt level and below. However, even with such high sensitivity, reliable detection of low-volatility vapors above the surface in real-world conditions remains a challenging task.

The main reason is that the vapor concentration above the soil surface is determined not only by the sensitivity of the analytical method, but also by complex interrelated processes occurring in the porous medium. After leaving the source, molecules are distributed among the gas, liquid, and solid phases of the soil, undergoing sorption, dissolution, diffusion, and biogeochemical transformation [5,6,7]. Consequently, the vapor concentration at the soil surface can vary by several orders of magnitude even with a constant source power [10,11,12]. Physicochemical studies of vapor in soil have shown that the equilibrium distribution between the phases and the kinetics of sorption processes can significantly limit the mobility of compounds in the soil and determine their availability for further transport [13]. The intensity of migration is influenced by porosity, permeability, pore space tortuosity, soil moisture, temperature, and meteorological conditions [3].

Numerous studies show that soil moisture is one of the key factors determining the transport of molecules. With changes in humidity, the surface flux of substance molecules can change by several orders of magnitude due to the redistribution of the substance between the gas, water, and sorbed phases [10]. According to the results of Pawłowski and Karpińska [14], TNT vapors were reliably detected over sandy samples, whereas for denser and more moisture-absorbing soils, their detection was significantly more difficult. Furthermore, drying of the surface soil layer can lead to a sharp increase in vapor sorption on mineral particles and, as a consequence, to a multiple decrease in the concentration of the substance above the soil surface [10,14].

A number of mathematical models have been developed to describe these processes. Early studies primarily used diffusion models to estimate the transport of chemical signatures through soil [15]. More complex models were subsequently developed that take into account the phase distribution of substances, sorption, degradation, and the transport of moisture and heat in porous media [10,16]. The developed numerical models made it possible to reproduce the influence of soil wetting and drying cycles on the flow of vapor to the surface [10]. Modern approaches to modeling the transport of substances in porous media utilize multicomponent transport equations based on the Darcy and Maxwell–Stefan laws, as well as reaction-transport models that take into account the interrelated processes of mass transfer and phase transformations [17,18,19]. However, despite the fact that the processes of dissolution, sorption, and phase distribution of substances in soil–water–vapor systems have been studied in sufficient detail [13], considerably less attention has been paid to the quantitative description of how the properties of the porous medium and its moisture content influence the migration of vapors from a buried source to the soil surface and, consequently, the possibility of their analytical detection. Currently, there are no experimentally validated models that allow for a quantitative relationship between soil properties (porosity, moisture content, pore space structure) and the vapor concentration of the substance being detected on the soil surface. The literature remains insufficient on the combinations of moisture content, porosity, and physical properties of the soil that allow vapors to reach the surface in concentrations sufficient for analytical detection [20]. As a result, a negative analytical result often cannot be unambiguously interpreted: it may indicate either the absence of a substance source in the soil or unfavorable mass transfer conditions.

Although the predictions of models such as those presented in the work of Raghunathan Ravikrishna et al. [21] are quite adequate for experiments on dry soil, they are inconsistent with those on wet soil. The influence of vapor diffusion in an aqueous medium must be taken into account so that the model can also describe vapor migration through wet soil to the surface. As demonstrated by experiment [14], in this case, the vapor density above the surface of wet soil is significantly higher than above the surface of dry soil. The rate of vapor diffusion in water is significantly (by 3–4 orders of magnitude) lower than in air. Nevertheless, vapor reaches the surface in much greater quantities.

Experimental observations show that vapor concentration above wet soil can exceed that above dry soil, despite the lower molecular diffusion of vapor in water. This behavior cannot be explained by traditional models of diffusion in dry soil and suggests that soil moisture affects not only the transport pathways but also the interaction of vapor with the soil.

Despite extensive research, existing models typically account for only a subset of transport mechanisms. Specifically, the combined effects of moisture-dependent sorption, reduced air-filled porosity, and pore blockage by small particles on vapor migration have not yet been quantitatively incorporated into a unified system.

The aim of this study is to develop a physics-based mathematical model for the molecular transport of low-volatility vapors in unsaturated porous media under realistic environmental conditions. We consider gas-phase diffusion, moisture- and temperature-dependent sorption, air-filled porosity, and pore blockage in the model.

Low-volatility energetic compounds were chosen as representative model substances because their extremely low vapor pressures provide rigorous testing for transport models. The model predictions were subsequently tested against laboratory experiments on several types of sand with different particle size distributions and physical properties. Experimental data published in [14] were also used.

Combining mathematical modeling and experimental data allows us to establish the conditions under which substance vapors can reach the soil surface and be detected by analytical methods, as well as to identify the fundamental limitations of approaches based on chemical signature detection.

The obtained results are important for the development of scientific understanding of the processes of mass transfer of trace organic compounds in porous media and their interaction with environmental factors, as well as for improving methods for detecting vapors of low-volatile substances above the soil surface.

## 2. Results and Discussion

### 2.1. Key Predictions of the Physics-Based Model

The physics-based continuum model developed in this work yields several quantitative predictions for the transport of low-volatility organic vapors through unsaturated porous media. The full mathematical formulation, including the governing equations, constitutive relations and numerical implementation, is presented in Section 3.1. Here we summarize the principal outcomes of the parametric analysis that are directly relevant to the experimental observations.

The model predicts a strongly non-monotonic dependence of the delayed effective diffusion coefficient on soil moisture, an optimum moisture content for surface vapor flux, a substantial reduction in transport caused by fine particles, and a pronounced temperature sensitivity. These predictions are examined below and subsequently compared with laboratory measurements.

**Figure 1:**
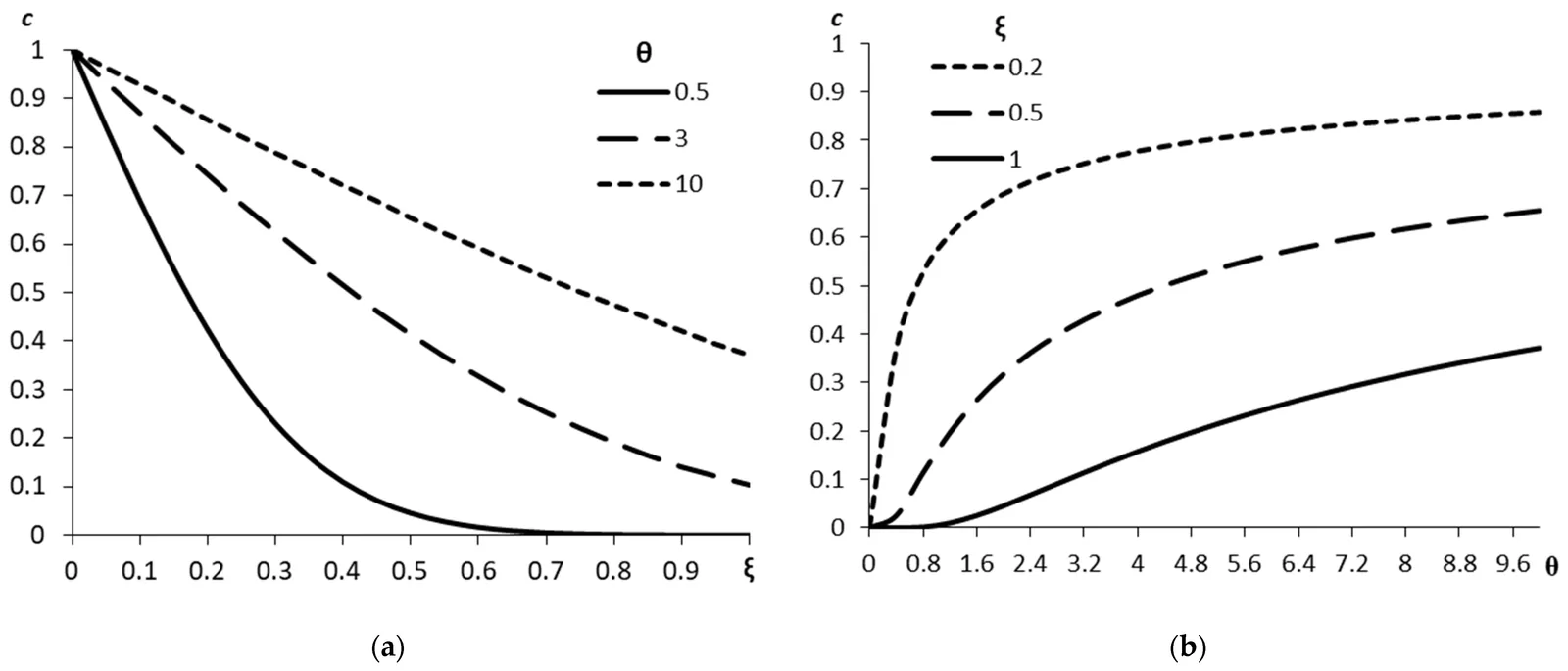
Dependence of dimensionless concentration on dimensionless depth (**a**) and on dimensionless time (**b**).

The distribution of the dimensionless concentration of a substance for different moments of time and different dimensionless distances from the source (calculated according to Equation (8), see Section 3.1) is shown in Figure 1.

As θ increases, the concentration of the substance in the soil becomes more uniform (Figure 1a). At θ ~ 1, it becomes significantly different from zero (0.005) in the surface layer. As time passes, the concentration of the substance in the soil increases, including in the surface layer (ξ = 1) (Figure 1b). The increase in concentration is initially rapid, starting from θ ~ 1, and then slows.

The scale *t_s_* is an important parameter in the dimensionless formulation. By estimating this scale, it is possible to approximately calculate the time at which the source of the explosive vapor can be detected. The parameter *t_s_* depends quadratically on the source depth and inversely on the diffusion coefficient *D*, which, in turn, is complexly determined by the diffusion rate of the vapor of a given substance in the soil.

We performed calculations for three model substances, the characteristics of which are presented in Table 1. We will model the propagation of substance molecules in sand. We adopt the following values: ρ*_T_* = 2.4 g/cm^3^, ρ*_b_* = 1.6 g/cm^3^, φ = 0.001 (dry sand), 0.11 (wet sand). The explosive characteristics are presented in Table 1.

The *K_sa_* parameter (at zero humidity) for all substances is taken from [21]: *K_sa_* (0) = 89,000 cm^3^/g. Note that such a large *K_sa_* value compared to *K_sw_* means that the adsorption of substance molecules from the air onto the soil surface is several orders of magnitude higher than adsorption from an aqueous medium.

**Figure 2:**
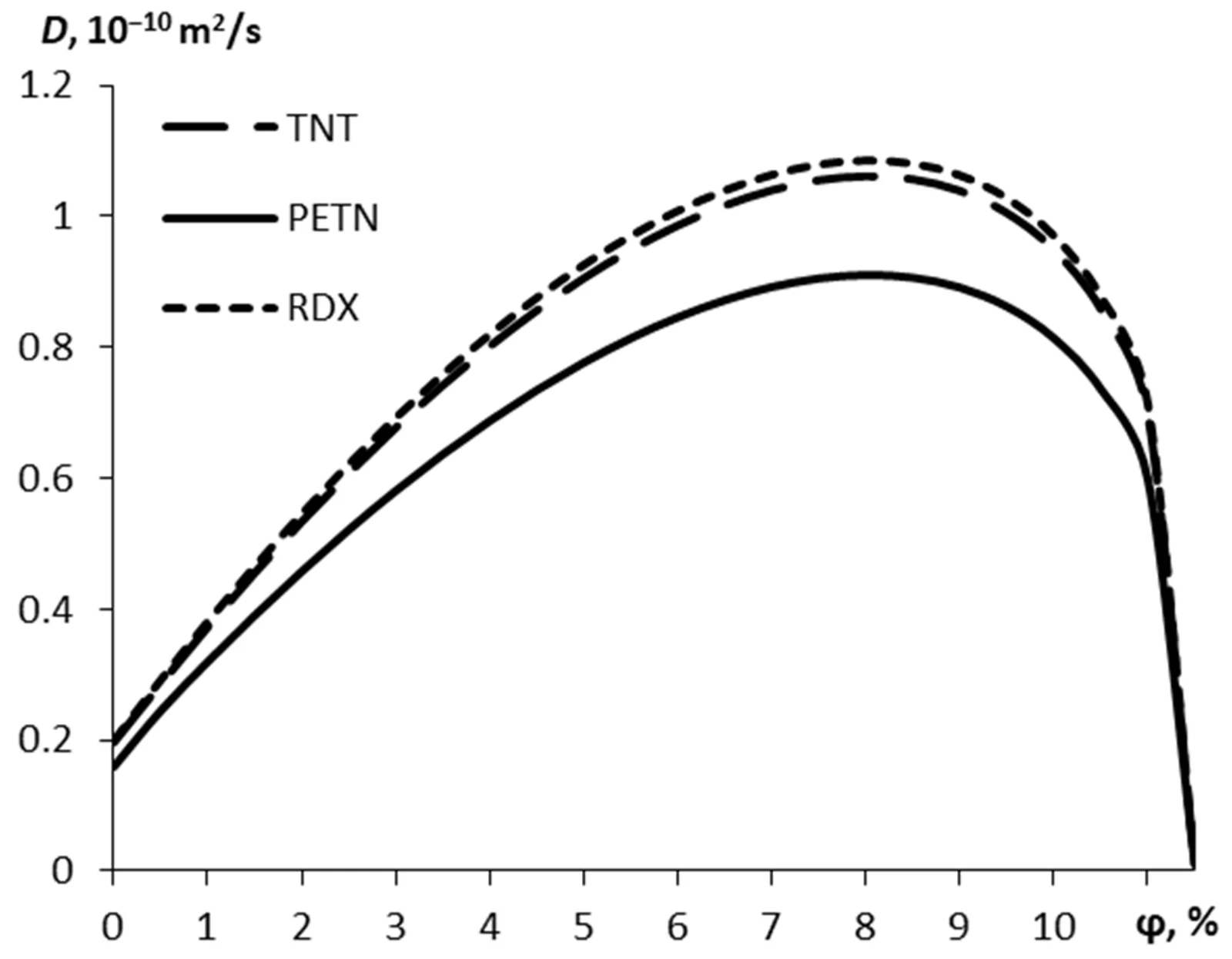
Delayed effective diffusion coefficient *D* depending on soil moisture.

Next, the effect of humidity on the effective delayed diffusion coefficient and the characteristic migration time of vapors through a sand layer is investigated. We assume ϕ_0_ = 0.01, *n* = 1. Figure 2 shows the dependence of the effective delayed diffusion coefficient of vapors on humidity.

A nonlinear relationship between the diffusion coefficient and soil moisture is observed. As the pore walls become wetter, the diffusion coefficient increases, reaching a maximum at ϕ*_cr_* ~ 7–8%, when the pore walls are completely covered with water and adsorption onto the soil solids is blocked. As the pores continue to fill with water, the diffusion coefficient initially decreases slightly, then approaches zero at the point of maximum soil moisture, at which air porosity becomes zero (the pores are completely filled with water, in which the vapors of the substance practically do not diffuse). In our calculation, this is ϕ > ϕ_max_ = 12%. Thus, at a certain low soil moisture content ϕ_cr_, conditions for vapor migration to the surface are created, but the vapors will be completely blocked in wetter soil at ϕ > ϕ_max_.

Calculation of the characteristic time scale ts shows that through a 5 mm layer, vapor in dry sand will travel to the surface within 20–25 h, and at a moisture content of 5 to 11%, within approximately 4 h. At higher moisture content, vapor will not travel to the surface for an extended period.

Since the diffusion coefficients in air for TNT and RDX are approximately the same, the curves in Figure 2 for these substances are very close. The curve for PETN is lower because it has a lower diffusion coefficient *D_a_*.

**Figure 3:**
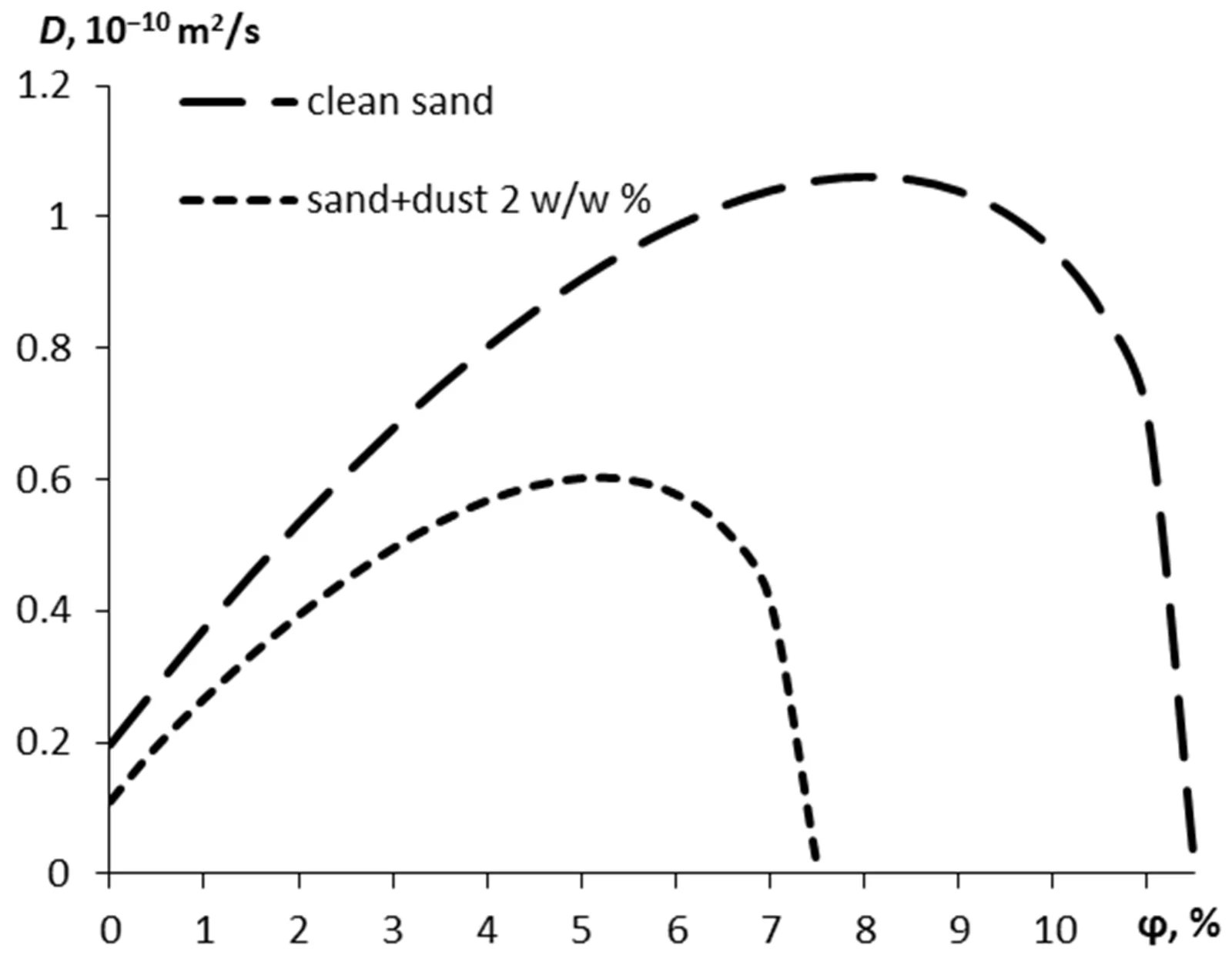
Delayed effective diffusion coefficient *D* depending on humidity: clean coarse sand and sand with fine dust fraction, 2 mass.%. Calculation for TNT.

Calculations show that adding 2% dust to coarse sand reduces the diffusion coefficient by approximately 44% in the air pores of dry sand. The effect of dust is reflected in a shift in the diffusion coefficient versus humidity curve toward zero (Figure 3, calculation for TNT). At lower ϕ_max_ values, the diffusion coefficient approaches zero, and vapor transport to the surface ceases, since the dust already occupies part of the pore volume. Furthermore, with increasing humidity, the diffusion coefficient increases significantly less than for pure sand.

**Figure 4:**
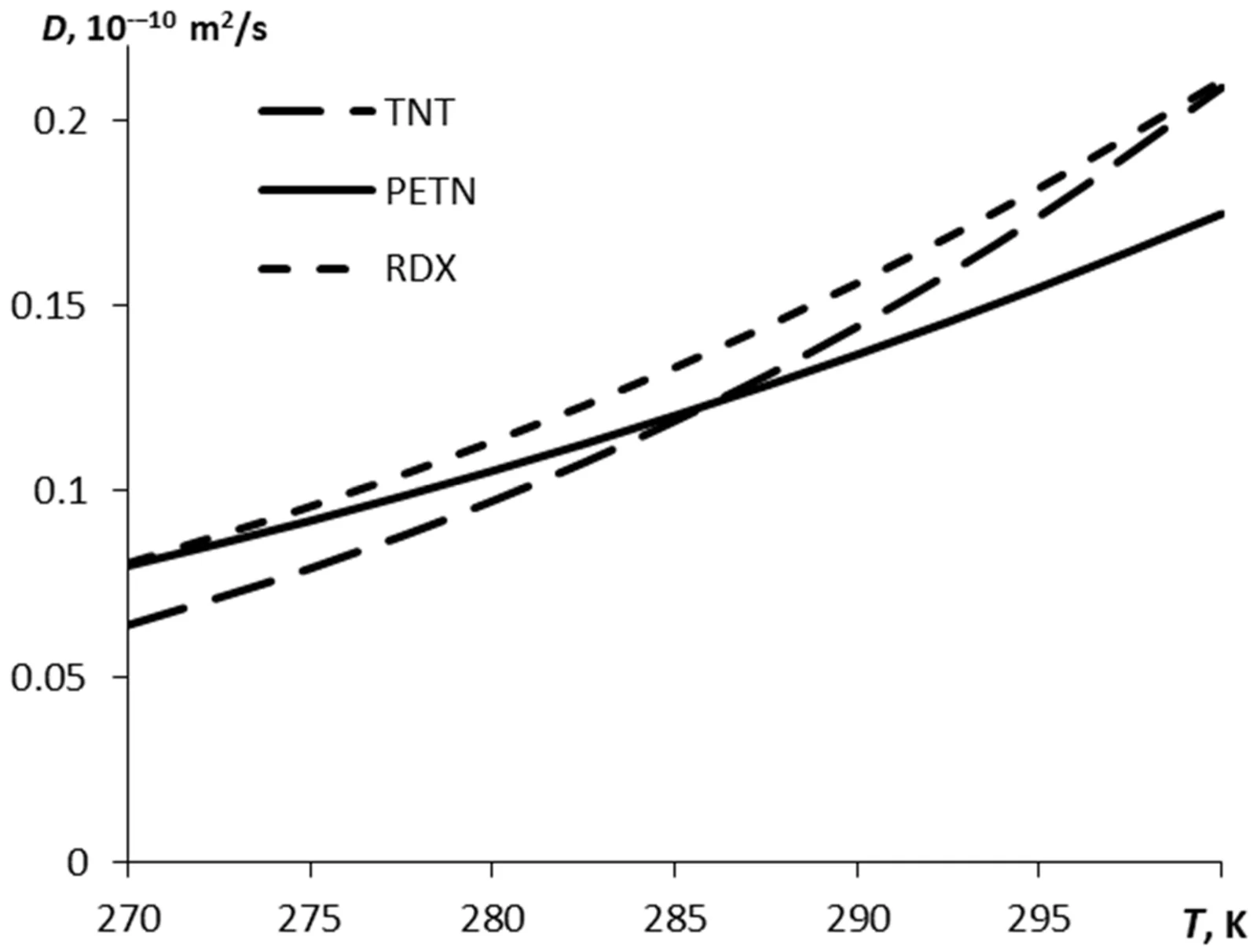
Delayed effective diffusion coefficient *D* depending on temperature.

Diffusion rate depends significantly on temperature. Considering the power-law dependence of the diffusion coefficient in air on temperature (power 3/2), expression (6) for the partition coefficient of physical sorption from the gas phase to the solid phase, and the data in Table 1, we calculated the diffusion coefficient *D* (delayed, effective) in dry sand for three model substances (Figure 4).

Compared to a temperature of 300 K, at 270 K, the diffusion coefficient drops by 60–70%. The rate of vapor migration to the surface is determined by the equivalent diffusion coefficient *D*, but the absolute value of the vapor reaching the surface depends not only on the burial depth, elapsed time, and migration velocity, but also on the vapor concentration at the source. The saturated vapor concentration for TNT and PENT is comparable in magnitude, but for RDX, it is 3–4 orders of magnitude lower. This means that even under identical soil moisture and temperature conditions, the surface concentration of RDX will be 3–4 orders of magnitude lower than that of TNT and PENT.

### 2.2. Experimental Validation

The experimental observations demonstrate a strong dependence of TNT vapor migration on the physical state of the sand pore space. Under dry conditions, a 1 cm layer of untreated sand containing the natural fine fraction (moisture content ≤ 0.1%) effectively suppressed vapor transport: no TNT vapor was detected in air samples above the sand during three weeks of observation. This behavior is consistent with the model prediction that fine particles reduce the connectivity of air-filled pores and substantially decrease the effective diffusion coefficient.

The transport behavior changed markedly after wetting the same sand. After three weeks of dry-sand observation, 1 mL of water was applied to the surface, producing a local moisture content of approximately 11%. TNT vapor was detected 55 min after wetting at a concentration of approximately 3 × 10^−13^ g cm^−3^. The concentration increased during approximately the first hour and subsequently decreased gradually. Thus, moderate moisture strongly enhanced vapor release compared with the dry dusty sand. This observation is consistent with the model mechanism in which water films reduce gas–solid sorption while a connected air-filled pore space is still preserved.

A similar qualitative behavior was observed for dust-free washed sand. For a 5 mm layer of washed dry sand (moisture content ≤ 0.1%), the TNT vapor concentration above the sand reached approximately 3 × 10^−14^ g cm^−3^ after several hours and remained approximately constant for 24 h. Increasing the layer thickness to 10 mm initially reduced the concentration by approximately 30%, followed by recovery within about 2 h. These observations indicate that removal of the fine fraction substantially facilitates gas-phase transport.

In contrast, high moisture content suppressed vapor migration. Spraying 3 mL of water onto a 4 cm layer of washed sand produced a moisture content of approximately 40% and reduced the TNT concentration at the surface to the background level. This result is consistent with the model prediction that, at sufficiently high moisture contents, water-filled pores disrupt the connected gas-phase transport pathways.

Temperature also had a pronounced effect. For a 1 cm layer of washed sand, decreasing the experimental temperature from 29 to 19 °C over 24 h reduced the recorded TNT vapor concentration to approximately three times the background level. This observation is consistent with the combined effects of reduced molecular diffusion and increased sorption at lower temperature.

**Figure A1:**
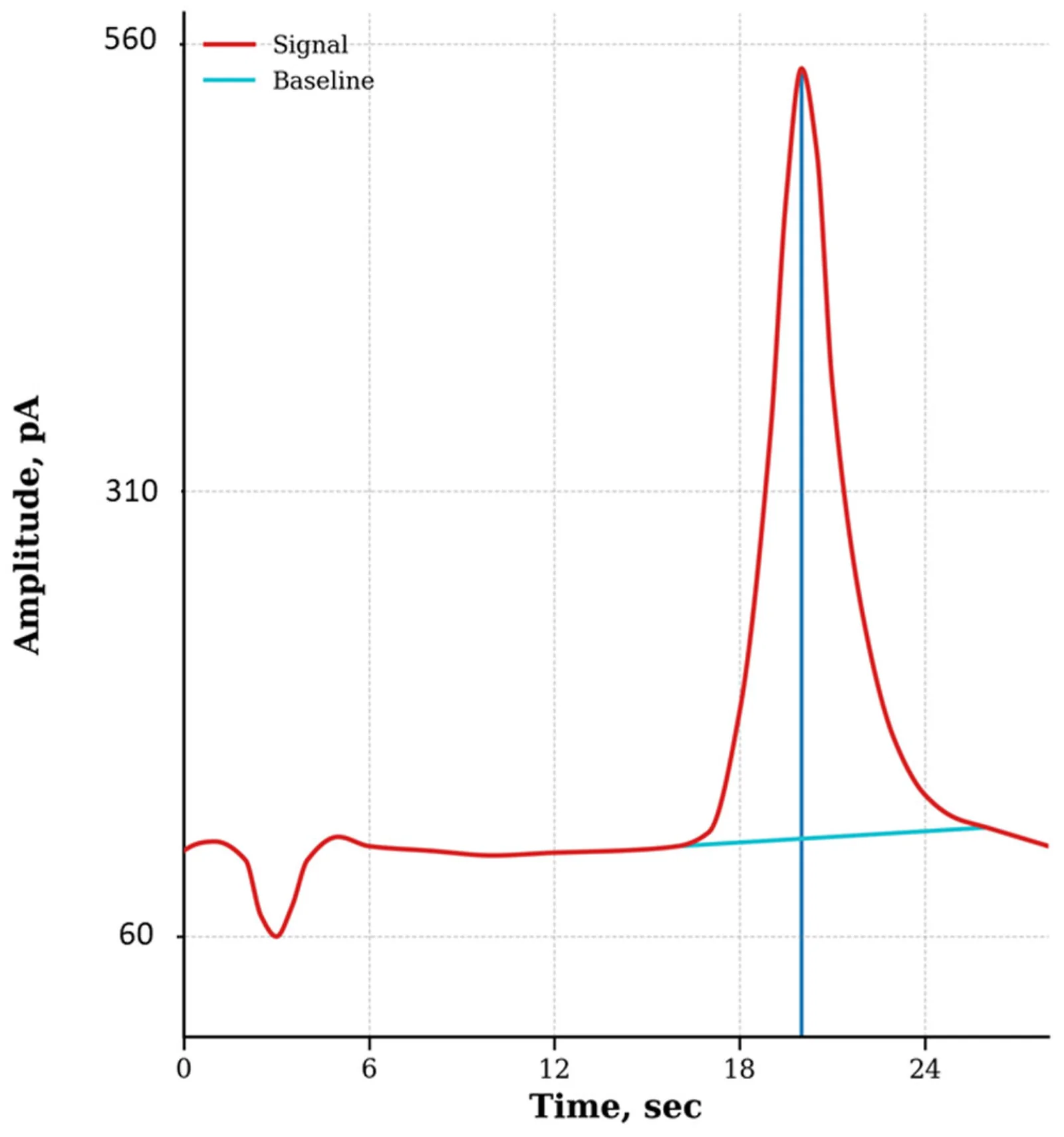
Chromatogram of a TNT vapor sample obtained from the vapor sensor at a concentration of 4.6 × 10^−14^ g/cm^3^. Sample volume: 100 mL; sampling rate: 500 mL/min; temperature: 20 °C. The blue vertical line indicates the peak value.

**Figure A2:**
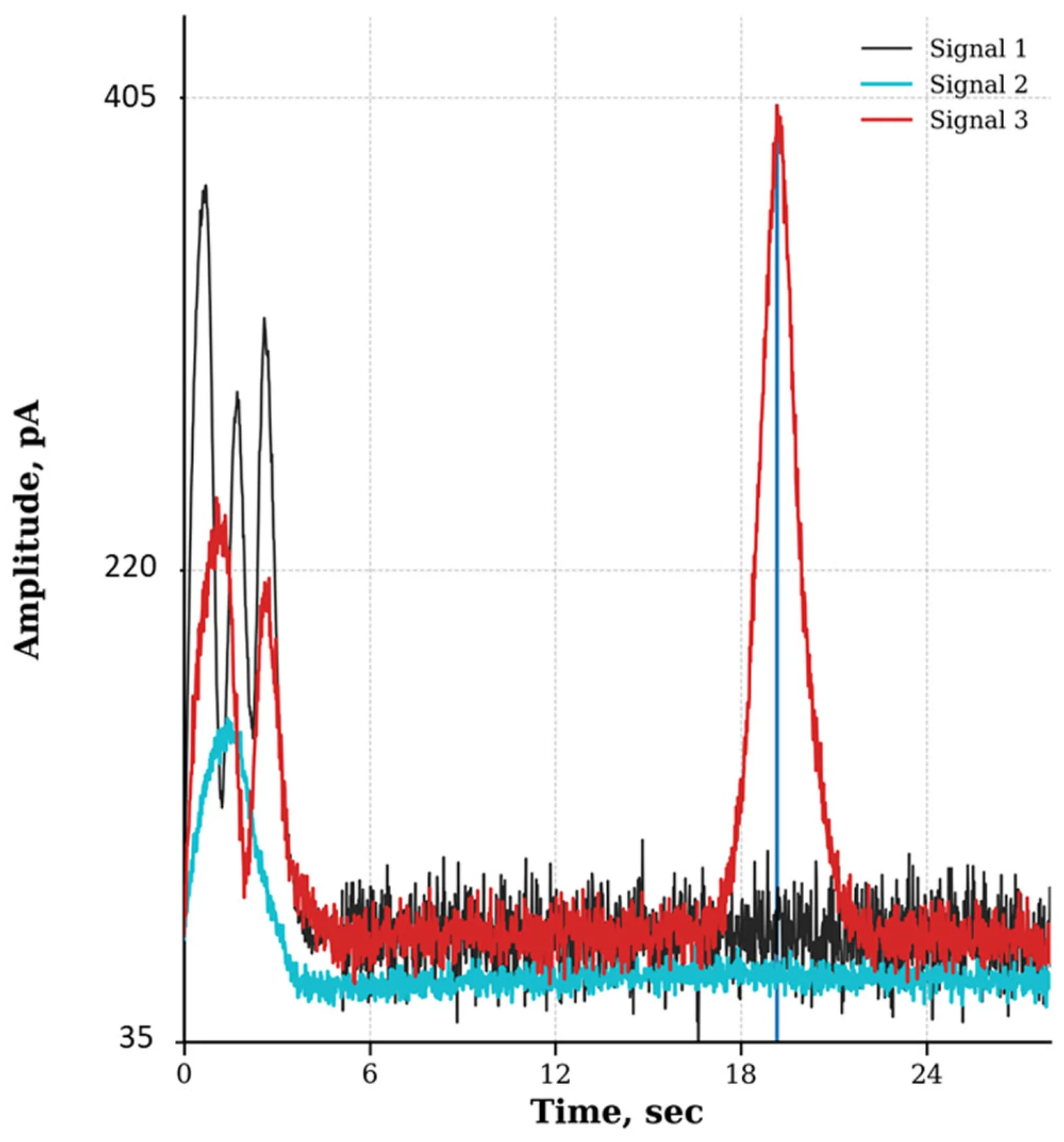
Effect of adding 1 mL of water: (1) chromatogram after water addition (concentration at the level of 1.6 × 10^−13^ g/cm^3^); (2) three chromatograms before water addition. The background and the signal above a 1 cm sand layer are at the noise level. The blue vertical line indicates the peak value.

The main experimental observations are summarized in Table 2. Representative chromatograms and the time dependence of the TNT vapor signal after wetting are provided in Appendix A (Figure A1 and Figure A2).

For each experimental condition, the TNT peak amplitude was determined from six replicate measurements. The estimated uncertainty of the concentration determination was approximately ±10–12%.

### 2.3. Comparison with Previous Experimental Studies

Our experimental and theoretical results are in qualitative agreement with the results of Pawłowski, W. & Karpińska, M. [14]. In that work, it was found that a 5 mm thick layer of dry sand effectively blocks the release of TNT vapors to the surface. In the experiment [14], an initial moisture level of the sand substrate was set, which then decreased as it dried. Both the moisture levels and the amount of TNT vapor above the surface were measured. Also, as predicted by our model and shown by our experiments, a nonlinear dependence of the TNT vapor level on the sand moisture content is observed. At a moisture content of approximately 2–3%, the maximum detectable vapor was observed (80–90 × 10^−13^ g/cm^3^), and at a higher moisture content—4–5%—a 4–5 times lower vapor level was recorded, as for almost-dry sand (φ < 1%). In our calculations, this result is obtained for sand containing a fine fraction (dust). An interesting result of the experiments is the detection of a second peak in the concentration of TNT vapor above the surface with a further increase in sand moisture content, at φ ~ 10–12% (and at higher moisture content, the amount of detected vapor drops to zero, which is consistent with our understanding of the “trapping” effect of water in sand pores). However, our model does not predict a second peak in relatively moist soil. The second peak at higher moisture content can be associated with the capillary rise of vapor with water to the surface, where it is released as the water evaporates. The authors explain [14]: “TNT partially dissolved in water is transported by the aqueous solution, moving via water films from one grain to another, to the surface.” The effect of the capillary rise of vapor substances in moist soil can be taken into account in further development of the model.

### 2.4. Physicochemical Mechanisms and Model Limitations

The obtained results demonstrate that vapor migration in unsaturated porous media is controlled by the competition between several coupled physicochemical processes rather than by molecular diffusion alone. The effective transport rate is determined by the balance between gas-phase diffusion, gas–solid sorption, and the evolution of the pore space as environmental conditions change. Because these mechanisms respond differently to variations in soil moisture, the resulting vapor flux is inherently non-monotonic.

At low moisture contents, adsorption on mineral surfaces substantially slows vapor migration because most adsorption sites remain available for interaction with vapor molecules. As moisture increases, thin water films progressively occupy these sites and reduce gas–solid interactions, thereby increasing the effective diffusion coefficient. However, further wetting decreases the volume and connectivity of the air-filled pore network available for gas transport. Consequently, moisture simultaneously promotes and suppresses vapor migration through different mechanisms, giving rise to an optimum moisture content at which the surface vapor flux reaches its maximum.

The model further indicates that fine particles influence vapor transport not only by reducing the effective pore diameter but also by modifying pore connectivity. This effect becomes particularly significant under partially saturated conditions, where transport pathways are already restricted by the presence of water. The combined action of moisture and pore clogging therefore cannot be represented by a simple correction to diffusivity but should be considered as a coupled structural effect governing the accessibility of continuous gas pathways.

Although the present study employs energetic compounds as representative low-volatility substances, the proposed framework is not restricted to this class of chemicals. The same physicochemical mechanisms govern the migration of many trace organic compounds emitted from subsurface sources, including environmentally relevant contaminants. Therefore, the developed model provides a general basis for predicting vapor transport in unsaturated porous media and may contribute to environmental monitoring, soil–atmosphere exchange studies, and other applications involving trace vapor migration.

From this perspective, temperature, moisture, and pore clogging should not be regarded as independent empirical factors. Instead, they represent coupled environmental variables that jointly determine the effective transport properties of the porous medium. The porous medium acts as an active regulator of molecular transport rather than a passive diffusion pathway.

It should be noted that the present continuum formulation does not resolve the pore-size distribution. While this simplification is adequate for the coarse sand examined here, the quantitative predictions may be less accurate for soils whose transport properties are dominated by a broad spectrum of micropores. Future work will incorporate measured PSDs or pore-network modeling to extend the applicability of the framework.

The continuum diffusion model deliberately neglects pore-scale geometric complexity and spatial heterogeneity. While this simplification allows the principal competing mechanisms (moisture-dependent sorption versus air-filled porosity) to be isolated and compared with laboratory data, it also defines the domain of applicability of the quantitative predictions. The model is most appropriate for homogeneous sandy media under intermediate moisture contents and for time scales on which gas-phase diffusion through continuous air pathways remains the dominant transport process. Extension to more complex soils will require incorporation of pore-network or dual-porosity descriptions.

The model parameters that control sorption are effective quantities that lump the effects of particle surface chemistry and mineralogy. While this approach is adequate for the relatively pure sand examined here, quantitative application to soils with substantially different surface properties (high clay content, abundant organic matter, or specific functional groups) would require re-determination or theoretical estimation of *K_sa_* and *K_sw_* for the particular solid matrix.

## 3. Materials and Methods

### 3.1. Theoretical Model Formulation

This section presents the complete formulation of the continuum model used to describe molecular transport of low-volatility vapors in unsaturated porous media. The model accounts for gas-phase diffusion, moisture- and temperature-dependent sorption, air-filled porosity and pore clogging by fine particles. The resulting analytical solution and the associated parametric study form the basis for the predictions reported in Section 2.1.

#### 3.1.1. Conceptual Framework of Vapor Transport

The conceptual picture developed below is based on extensive experimental and theoretical evidence for the moisture dependence of vapor-phase sorption and diffusion in unsaturated soils. In dry porous media, mineral surfaces act as strong adsorbents for organic vapors. Even modest increases in water content lead to competitive displacement of organic molecules by water and progressive coverage of mineral surfaces by thin water films, thereby strongly suppressing gas–solid sorption [29,30,31]. At intermediate moisture contents, the residual air-filled pore network remains connected, so that the reduction in delay outweighs the modest decrease in air-filled porosity and vapor transport is enhanced. Further wetting reduces air-filled porosity and eventually disconnects continuous gas pathways, suppressing gas-phase diffusion [32]. Fine particles occupying the pore space further restrict connectivity and increase specific surface area available for sorption. These competing effects produce a non-monotonic dependence of vapor flux on soil moisture.

**Figure 5:**
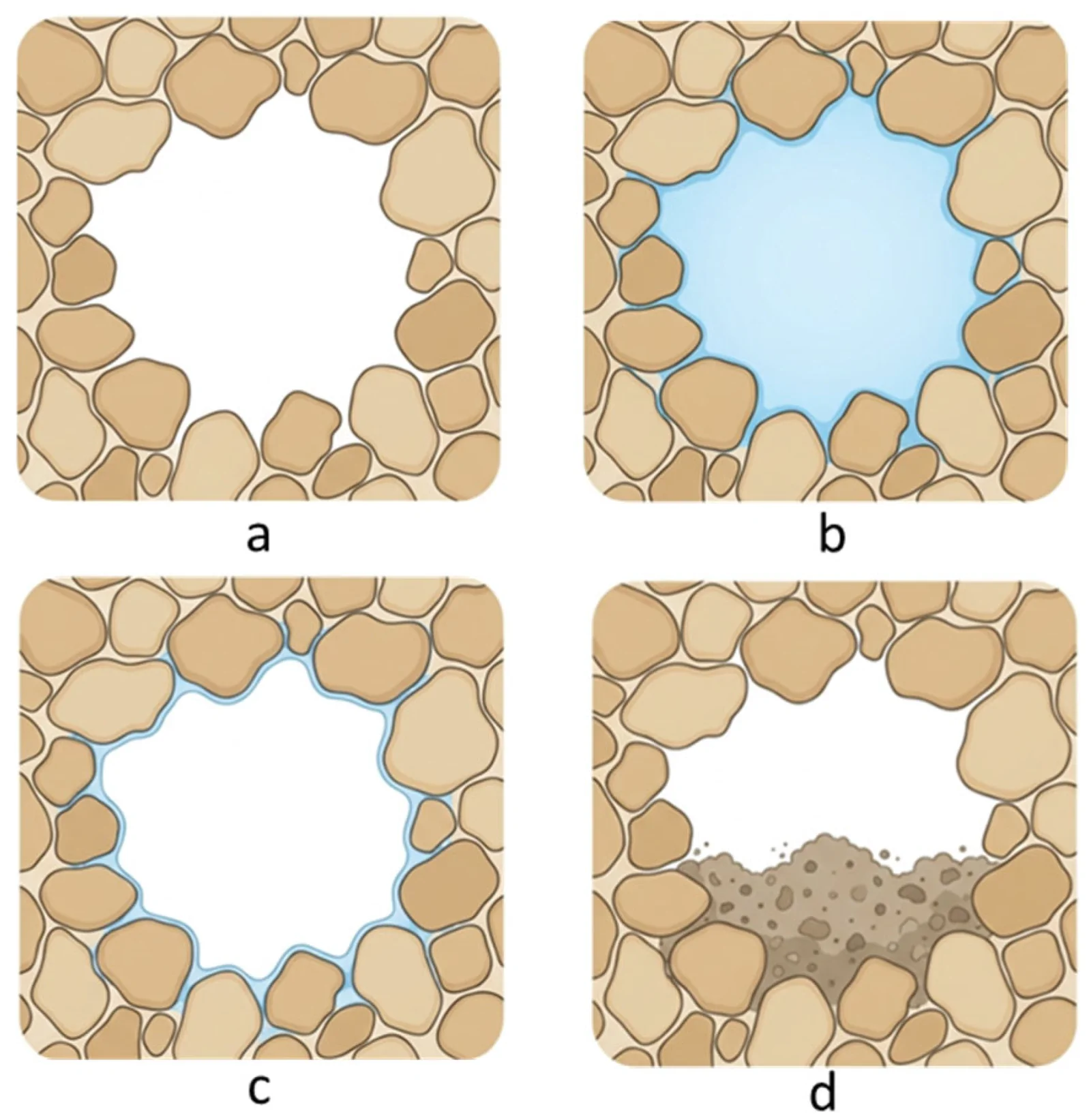
Diagram of the state of pores in the soil depending on the distribution of water in the pores: dry pores filled with air (**a**), pores filled with water (**b**), pores covered with a film of water (**c**), pores partially filled with a fine dust fraction (**d**).

The transport of explosive vapors in soil is determined by the physical state of the pore space. Soil moisture exerts a significant influence. Depending on the water distribution, four characteristic pore states can be identified (Figure 5a–c), each associated with specific processes of explosive molecule transport.

1. Dry air-filled pores (Figure 1a). Immediately after vapor release, explosive molecules are predominantly adsorbed onto mineral surfaces. During this transient stage, adsorption acts as a strong sink, resulting in extremely slow propagation of the vapor front. Once the available sorption sites approach equilibrium, further transport proceeds mainly by gas-phase diffusion through interconnected air-filled pores, while adsorption and desorption remain locally balanced and no longer significantly retard long-range migration.
2. Water-filled pores (Figure 5b). Pores completely occupied by water contribute little to gas-phase transport. Because diffusion of nitroaromatic vapors in water is two to three orders of magnitude slower than in air (as will be shown in greater detail below), and because Henry’s constants are very small, transport through these pores is negligible on the time scales considered. Such pores therefore effectively interrupt gas diffusion pathways.
3. Water-film-covered pores (Figure 5c). At intermediate moisture contents, thin water films cover mineral surfaces while pore centers remain air-filled. Under these conditions, mineral adsorption sites become progressively screened by water. Vapor molecules are therefore much less strongly retained by the solid matrix and diffuse primarily through the remaining air-filled pore space. Immediately after wetting, displacement of previously adsorbed molecules may produce a transient increase in vapor concentration, whereas after equilibrium is established, diffusion through the free pore space becomes the dominant effect, accompanied by a decrease in sorption at the water–solid interface.
4. Partially water-filled pore networks. At higher moisture contents, some pores become completely water-filled whereas others remain only film-coated. The resulting transport behavior combines reduced sorption with progressive loss of gas connectivity. Consequently, vapor migration first increases with moisture because adsorption weakens, but eventually decreases as continuous air pathways disappear.

These four regimes are consistent with the dual-sorbent concept of organic vapor uptake in unsaturated soils [29,30] and with experimental observations of enhanced vapor detection at intermediate moisture levels [14].

Thus, soil moisture affects explosive vapor transport through two competing mechanisms. Increasing moisture progressively suppresses gas–solid sorption by covering mineral surfaces with water films, thereby increasing the effective diffusion coefficient. At the same time, continued wetting decreases air-filled porosity and eventually disconnects the gas-phase transport pathways. The competition between these two mechanisms is hypothesized to produce the experimentally observed non-monotonic dependence of surface vapor concentration on soil moisture.

In addition to moisture distribution, the geometry of the pore network also controls vapor transport (Figure 5d). Fine mineral particles (dust) occupying the pore space reduce the volume and connectivity of air-filled pathways, thereby decreasing the effective diffusion coefficient. Unlike soil moisture, which primarily modifies vapor transport through changes in gas–solid sorption and air-filled porosity, dust acts predominantly through structural restriction of the pore network and a substantial increase in the surface area available for the adsorption of explosive molecules. These two effects are treated independently in the present model.

This conceptual picture forms the physical basis of the mathematical model developed below.

#### 3.1.2. Problem Formulation

**Figure 6:**
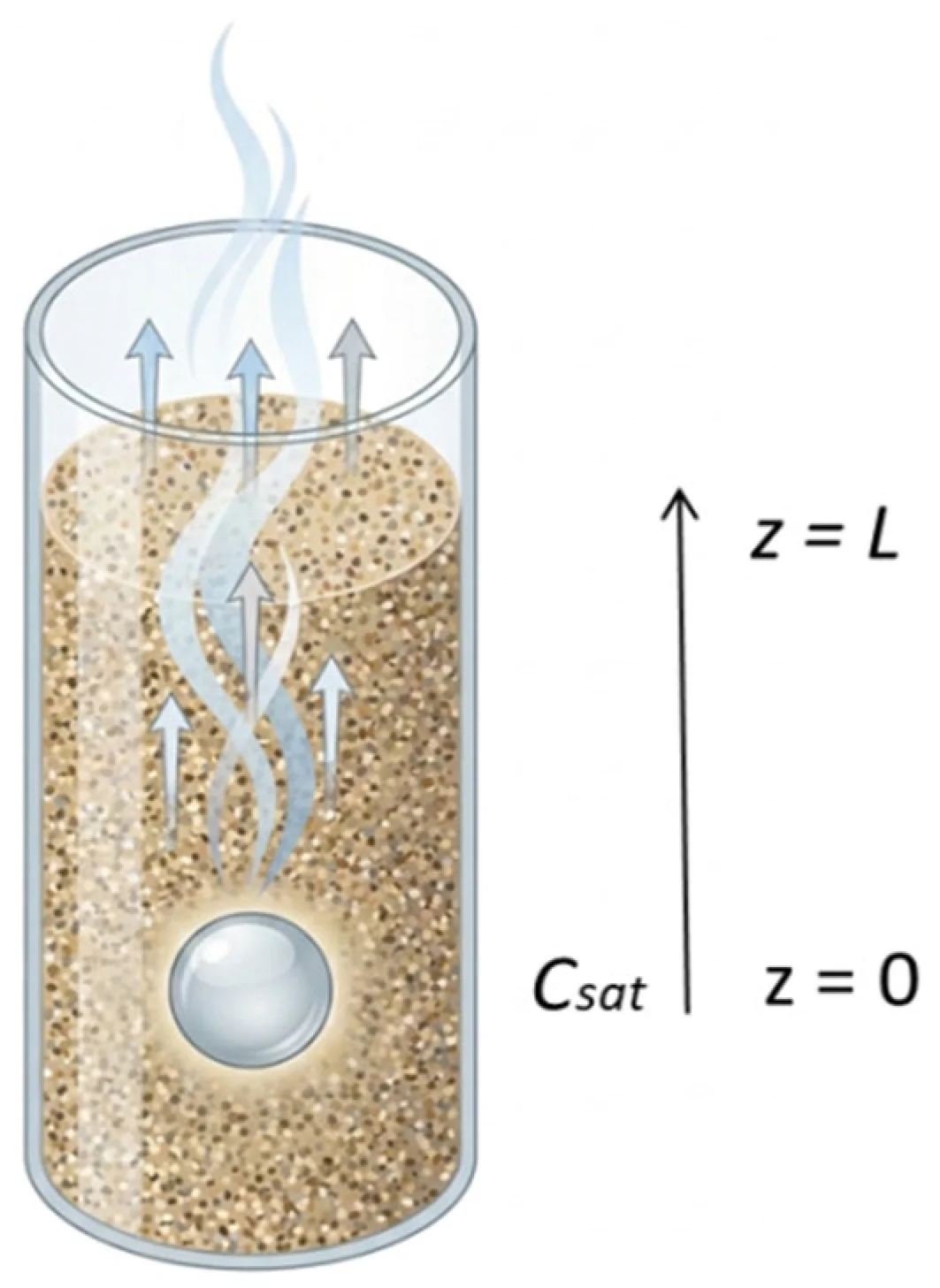
Schematic diagram of the problem of migration of vapors of a model substance from a source in the soil.

At a depth of *L* in the soil, a source of a model substance is located for a time *t* (Figure 6). The soil is characterized by moisture content, density, and porosity. The substance is characterized by the saturated vapor density *C_sat_*, the vapor diffusion rate in air *D_a_*, the vapor diffusion rate in water *D_w_*, and the constants of interaction with soil and water (Henry’s constant *H*, etc.). The goal is to determine the spatiotemporal distribution of vapor concentration *C*(*z*,*t*) in the soil in a one-dimensional formulation and to predict the concentration above the soil surface *C_s_*(*t*) = *C*(*L*,*t*), which is compared with the results of gas chromatographic analysis.

#### 3.1.3. Model Assumptions

The following assumptions were made when constructing the mathematical model.

1. The model substance is characterized by low solubility in water and low Henry’s law coefficients for water-vapor environments. An explosive is considered the model substance [21].
2. The primary mechanism of mass transfer is molecular diffusion. The effective diffusion coefficient is determined by air- and water-filled porosity. Due to the small diffusion coefficients in water, the contribution of transport through the aqueous phase is negligible [21,27].
3. Sorption on the surface of soil particles is considered a process that slows diffusion and is accounted for by the delay coefficient *R_f_*, which depends on soil moisture [30].
4. The gas and liquid phases are in local thermodynamic equilibrium. The distribution of the substance between them is described by Henry’s law, which allows the use of a single effective transport coefficient [21].
5. Biodegradation. It is assumed that the characteristic biodegradation time significantly exceeds the diffusion transfer time; therefore, the influence of this process can be neglected [30].

The model is formulated at the continuum (REV) scale and therefore employs only macroscopic effective parameters. Spatial heterogeneity of the pore network, local capillary geometry and pore-scale connectivity are not resolved. Consequently, the present formulation is expected to be most reliable for relatively homogeneous, coarse-textured media (such as the sand used in the laboratory experiments) and for short transport distances and times. Application to structured or fine-textured soils would require additional information on pore-size distribution and preferential pathways.

#### 3.1.4. Basic Equations

The total concentration of soil molecules migrating through both air and water pores (the concentration in the soil pore space): *C* = (ε*_w_C_w_* + ε*_a_C_a_*)/ε_T_, where ε*_a_* is the air-filled porosity, ε*_w_* is the water-filled porosity, and ε*_T_* is the total porosity.

The water-filled, air-filled, and total porosity of the soil can be determined as follows, given the true density of the soil material ρ*_s_*, the water density ρ*_w_*, and the bulk density of the soil ρ*_b_*: $\varepsilon_{T}=1-\frac{\rho_{b}}{\rho_{s}}\left(1+\varphi \right)=\varepsilon_{a}+\varepsilon_{w}.$ The total porosity of the soil varies between 0.25 and 0.80. General considerations suggest that water-filled porosity is related to the bulk soil moisture φ by the formula: $\varepsilon_{w}=\varphi \frac{\rho_{s}}{\rho_{w}},$ $\varepsilon_{a}=1-\frac{\rho_{b}}{\rho_{s}}-\varphi \left(\frac{\rho_{b}}{\rho_{s}}+\frac{\rho_{s}}{\rho_{w}}\right).$

Soil porosity with a dust fraction. A typical case is soil composed of coarse particles (e.g., coarse sand) and fine dust, which can fill the soil pores, reducing its porosity. If the relative mass of such dust is *m_dust_*, then the air-filled porosity is expressed as follows: $\varepsilon_{a}=\varepsilon_{a0}-m_{dust}\frac{\rho_{b}}{\rho_{dust}},$ where ε*_a_*_0_ is the air porosity of the dust-free soil, and ρdust is the density of dust particles. For example, adding ~10% by weight of dust to coarse washed sand reduces its air porosity by approximately 5–10 percentage points (depending on the density of the dust). Conversely, if the sand is free of dust, its air porosity increases.

Equation for the concentration of the vapors in a soil layer at a distance *z* from the source:

(1) $$\frac{\partial C}{\partial t}=D\frac{\partial^{2}C}{\partial z^{2}}-k_{d}C,$$

where *D* is the diffusion coefficient slowed down by sorption in the solid phase: *D* = *D_eff_*/*R_f_* (hereinafter we will call it the “diffusion coefficient” or “delayed effective diffusion coefficient”); *k_d_* is the biodegradation reaction parameter.

With initial condition: *t* = 0, *z* > 0, *C* = 0;

With boundary conditions:

*z* = 0 (vapor source location point):

*C* = *C*_0_ = (ε_w_*C_w_*_0_ + ε_a_*C_sat_*)/ε_T_,

*C_w_*_0_ = *C_sat_* · *H*,

*C_sat_*—concentration of saturated vapors of a substance.

z=∞:C=0.

For *H* << 1, we take *C_w_* = 0.

For explosive vapors, the characteristic value of the Henry coefficient in relation to water is 10^−6^ or less [22,23].

To estimate the effective diffusion coefficient, we use the Millington–Quirk equation [27]:

(2) $$D_{eff}=D_{a}{\left(\frac{\varepsilon_{a}}{\varepsilon_{T}}\right)}^{10/3}+D_{w}{\left(\frac{\varepsilon_{w}}{\varepsilon_{T}}\right)}^{10/3}.$$

For explosive vapors, the diffusion coefficient in water is typically 2–3 orders of magnitude smaller than the diffusion coefficient in air [33]. Therefore, the second term in Equation (2) can be neglected.

The retardation coefficient *R_f_* is a dimensionless factor reflecting how much slower a substance spreads in a porous medium compared to diffusion in air, due to its retention in the solid phase (adsorption). Since vapor migration occurs in both air- and water-filled pores, the retardation coefficient is expressed by the sum [34]:

(3) $$R_{f}=\left(\varepsilon_{w}R_{fw}+\varepsilon_{a}R_{fa}\right)/\varepsilon_{T},$$

where $R_{fw}=1+\frac{\rho_{s}}{\varepsilon_{w}}K_{sw}$, $R_{fa}=1+\frac{\rho_{s}}{\varepsilon_{a}}K_{sa},$ *K_sw_* is the sorption (distribution coefficient) from the aqueous phase to the solid phase (m^3^/kg), *K_sa_* is the adsorption (distribution coefficient) from the gas phase to the solid phase (m^3^/kg), ρ_s_ is the density of dry soil.

Important parameters of the problem are the partition coefficients from the gas phase to the solid phase and from the aqueous phase to the solid phase, *K_sa_* and *K_sw_*. The following estimate can be made for *K_sw_*, using the soil organic fraction *f_oc_* and the organic matter distribution [33]:

(4) $$K_{sw}=K_{os}\cdot f_{os},$$

where *K_os_* is the sorption coefficient for organic matter, and *f_os_* is the mass fraction of organic carbon in the soil (usually from 0.001 to 0.05).

The sorption coefficient from the gas phase into the soil through pores covered with a layer of water molecules is difficult to determine. The effect of moisture on the sorption coefficient from an air-filled pore into the soil can be determined as follows [35]:

(5) $$K_{sa}(\varphi )=(1-S(\varphi ))K_{sa}(0)+S(\varphi )H\varphi ,$$

where *S*(ϕ) is a function describing the degree of pore surface coverage with water, and ϕ is the volumetric soil moisture content. This is a semi-empirical function reflecting the fraction of the pore surface covered by water.

Introducing the function *S*(ϕ) allows us to take into account the influence of soil moisture on vapor transport to the surface. We use the following form of the function [34]: $S(\varphi )=\frac{\varphi^{n}}{\varphi^{n}+\varphi_{0}^{n}},$ where *n* and ϕ_0_ are empirical constants. For moist soil, *S*(ϕ) → 1. Then, sorption is described by gas dissolution in water (the second term of Equation (5)). *H*·ϕ is the equivalent volume of the sorbed substance dissolved in water. For dry soil, *S*(ϕ) → 0, and *K_sa_* = *K_sa_*(0); that is, it corresponds to sorption in dry soil.

In the present continuum formulation, the chemical and physical characteristics of the soil particles (mineral composition, surface functional groups, specific surface area) are not resolved explicitly. Their influence is embodied in the effective partition coefficients *K_sa_*(0) and *K_sw_*. For the low-organic-carbon quartz sand used in the experiments, the large value of *K_sa_*(0) reflects predominantly physical adsorption on mineral surfaces; the rapid decline of *K_sa_* with increasing moisture is described by the empirical coverage function *S*(φ).

Temperature dependence: the temperature dependence of the coefficient of physical sorption from the gas phase to the solid phase is exponential (Arrhenius) and is described by the equation [24]

(6) $$K_{sa}(T)=K_{0}\exp\left(\frac{Q}{RT}\right),$$

where *Q* is the heat of sorption, *K*_0_ is the pre-exponential factor (entropy factor), *R* is the universal gas constant, and *T* is the absolute temperature.

The diffusion coefficient also depends on temperature. In air, the diffusion coefficient *D_a_* ~ T^3/2^, *D_w_* increases according to the Arrhenius law (however, although this coefficient increases with temperature, it is still 1–3 orders of magnitude smaller than the diffusion coefficient in air).

Analytical solution of Equation (1):

(7) $$C(z,t)=C_{0}e^{-k_{d}t}\mathrm{efrc}\left(\frac{z}{\sqrt{4Dt}}\right).$$

By introducing the natural scales of distance—*L*, time—$t_{s}=\frac{L^{2}}{16D}$, we obtain Equation (7) in dimensionless form:

(8) $$C(z,t)=C_{0}e^{-k_{d}t}\mathrm{efrc}\left(\frac{z}{\sqrt{4Dt}}\right).$$

Here, θ is *t*/*t_s_*, *c* = *C*/*C*_0_ is the dimensionless concentration (the ratio of the vapor concentration to the concentration at the mine’s location), and ξ = *z*/*L*. The dimensionless variables *c* and ξ range from 0 to 1, $K_{d}=k_{d}t_{s}=\frac{L^{2}k_{d}}{16D}.$ The physical meaning of the parameter *t_s_* is the time it takes for the substance to reach its maximum concentration on the soil surface. The *K_d_* complex is the ratio of the characteristic time of vapor migration to the surface to the characteristic time of biodegradation. In our problem, we assume that biodegradation effects are much longer lasting than the effects of vapor transport to the surface. Therefore, we assume *K_d_* ~ 0.

All calculations based on the analytical solution (Equations (7) and (8)) and the expressions for the moisture- and temperature-dependent delayed effective diffusion coefficient *D* were performed in Microsoft Excel (Microsoft 365) using the built-in complementary error function and standard worksheet arithmetic. No external numerical libraries or proprietary codes were employed.

### 3.2. Experimental Materials and Procedures

#### 3.2.1. Materials

We chose construction-grade sand (GOST 8736-2014 [36]) as our soil model. This sand has a maximum particle size of 1.2 mm and a high dust content. We believe this soil is the simplest model for studying vapor migration processes, taking into account various factors. We used the original sand, dried to a moisture content of no more than 0.1% and dedusted. Moisture content was adjusted by adding the required mass of water relative to the mass of dry sand.

Dust fraction removal (dedusting) was accomplished by repeatedly rinsing the sand in clean tap water. It was found that the mass of the dust fraction accounted for approximately 2% of the sand mass.

The sand is a relatively coarse, well-sorted material (maximum particle size 1.2 mm). A detailed pore-size distribution was not measured; the continuum model employed in this study uses bulk air-filled and total porosities rather than an explicit PSD.

**Figure 7:**
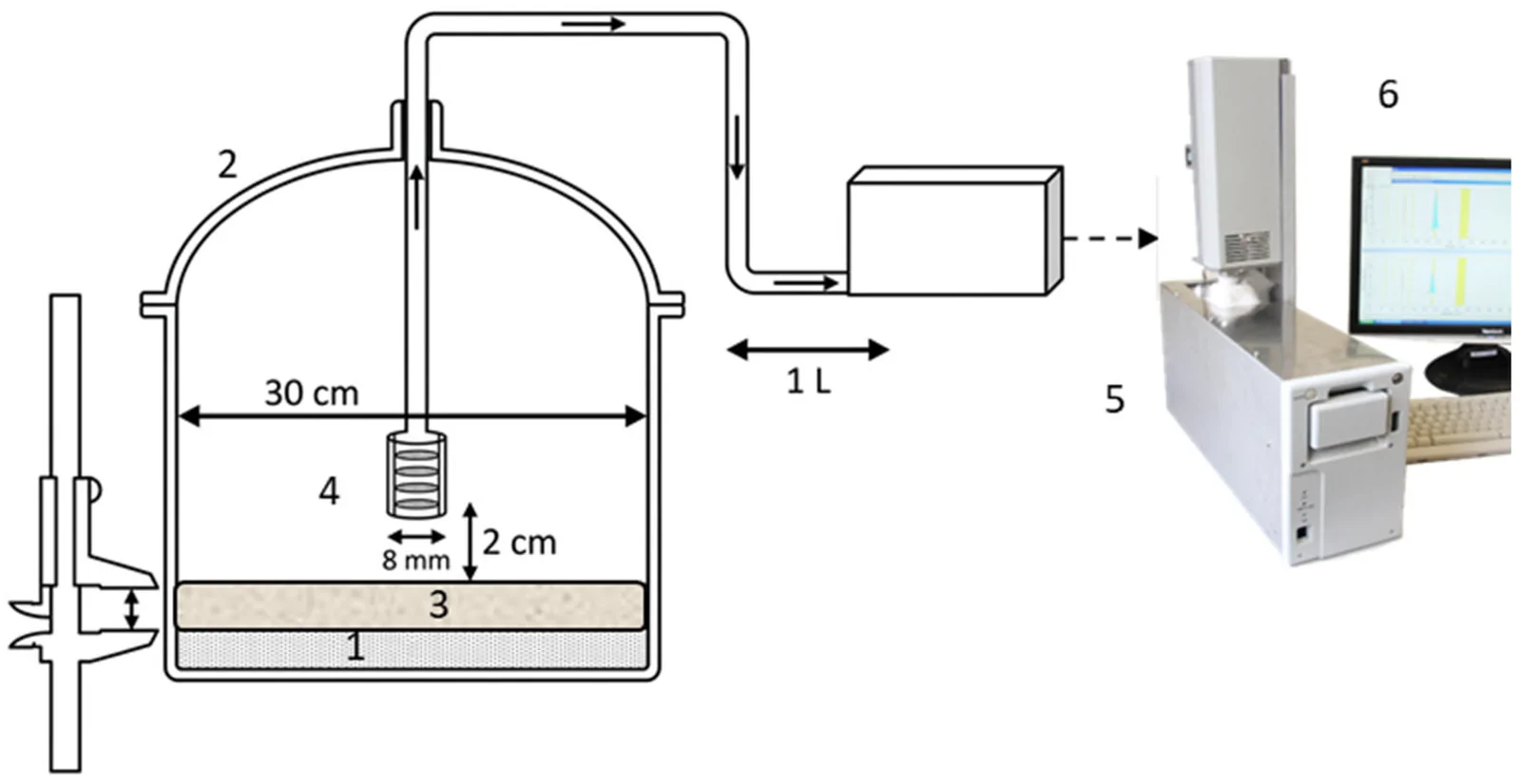
Diagram of the explosive simulator installation and sampling: TNT simulator (1), desiccator (2), sand layer (3), concentrator (4), gas chromatograph (5), computer (6).

The diagram of TNT vapor loading and sampling is shown in Figure 7. A 200 g TNT simulant (GOST 4117-78 [37]) manufactured by GosNII Kristall (Dzerzhinsk, Russia) (item 1) was used as a vapor source. The dimensions of the simulant were 100 × 50 × 25 mm^3^. The simulant was placed on the bottom of a glass jar, a desiccator (item 2). The average diameter of the desiccator with a lid was 30 cm. The simulant was covered with sand (item 3). The thickness of the sand layer above the simulant was measured with a caliper. Vapor samples from a distance of 2 cm above the ground, as shown in Figure 7, were collected on a concentrator by direct suction using a POU-I sampling device (ITs. 807.Sb0101-100 RE) (JSC Production Association “Elektrototchpribor”, Omsk, Russian Federation). An 8 mm diameter concentrator made of 4 metal meshes was placed at the end of the tube (item 4). A sample volume of up to 1 L was passed through the concentrator at a constant speed, ensuring the complete capture of TNT vapors [38].

#### 3.2.2. Methods

To record trace concentrations of TNT vapor, a portable polycapillary express gas chromatograph (GC) ECHO-SPIP (item 5) with a detector in the form of an ion mobility increment spectrometer (IMIS), manufactured by the Trofimuk Institute of Petroleum Geology and Geophysics of Siberian Branch Russian Academy of Sciences (Novosibirsk, Russian Federation), was used. This GC is characterized by a high threshold sensitivity of 10^−16^ g/cm^3^ when collecting 1 L of sample per concentrator [38]. The reaction time is up to 30 s. The chromatograph is connected to a personal computer (item 6). Sample introduction from the concentrator is carried out by the thermal desorption method. Control of the GC operating modes and analysis of samples from the concentrator is carried out by special software. The sensitivity of the ECHO-SPIP GC was monitored using a TNT vapor concentration sensor (VCS) of 4.6 × 10^−14^ g/cm^3^ at a temperature of 20 °C.

Analytical measurements and repeatability. TNT vapor concentration at the sand surface was estimated from the amplitude of the TNT peak in the chromatogram. For each experimental condition, the peak amplitude was determined from six replicate measurements. The estimated measurement uncertainty/repeatability of the analytical determination was approximately ±10–12%. TNT vapor was measured using a portable polycapillary express gas chromatograph (ECHO-SPIP) equipped with an ion mobility increment spectrometer detector. The threshold sensitivity of the analytical system was ≤10^−16^ g cm^−3^ for a 1 L air sample. The sensitivity of the chromatograph was monitored using a TNT vapor concentration sensor (4.6 × 10^−14^ g cm^−3^ at 20 °C). Samples were collected 2 cm above the sand surface by direct suction, with up to 1 L of air passed through the concentrator. The concentrator had a diameter of 8 mm and consisted of four metal meshes. Sample introduction was performed by thermal desorption. The chromatographic column consisted of 990 capillaries with a diameter of 40 μm and a SE-30 stationary phase (0.2 μm); the column and detector temperatures were 155 °C and 120 °C, respectively.

## 4. Conclusions

A physics-based model describing the transport of low-volatility organic vapors in unsaturated porous media has been developed and experimentally validated. Unlike previously proposed approaches, the model explicitly accounts for the coupled effects of moisture-dependent sorption, reduction in the air-filled pore space, temperature, and pore clogging by fine particles, allowing the influence of environmental conditions on vapor migration to be evaluated within a unified physicochemical framework.

The model demonstrates that vapor transport is governed by the competition between several coupled mechanisms rather than by molecular diffusion alone. Increasing soil moisture initially enhances vapor migration by reducing gas–solid sorption through the formation of thin water films on mineral surfaces. At higher moisture contents, however, the progressive loss of air-filled pore connectivity suppresses gas-phase diffusion. The resulting balance between these opposing effects produces a non-monotonic dependence of the surface vapor flux on soil moisture, with an optimum corresponding to the maximum transport efficiency.

The developed model further shows that pore clogging by fine particles substantially decreases the effective transport capacity of porous media by restricting continuous diffusion pathways, whereas lower temperatures suppress vapor migration through the combined effects of reduced molecular diffusivity and stronger sorption. These results demonstrate that the physicochemical state of the porous medium plays a decisive role in determining whether vapors generated by a subsurface source can reach the ground surface.

Experimental studies using TNT as a representative low-volatility compound confirmed the principal qualitative predictions of the model, including the existence of an optimum moisture content and the strong influence of pore structure and temperature on vapor transport. The agreement between theoretical predictions and experimental observations supports the applicability of the proposed approach for describing molecular transport under realistic environmental conditions.

Although energetic compounds were employed as representative model substances because of their extremely low volatility, the proposed framework is applicable to a much broader class of trace organic compounds emitted from subsurface sources. Consequently, the developed model provides a general physicochemical basis for predicting vapor transport in unsaturated porous media and may be useful in environmental monitoring, soil–atmosphere exchange studies, contaminant migration analysis, and other applications involving trace vapor transport through porous materials.

More generally, the present study demonstrates that an unsaturated porous medium should be regarded not as a passive diffusion pathway but as an active regulator of molecular transport whose physicochemical state controls the probability that vapor molecules generated at depth will reach the surface.

## Figures and Tables

**Table 1:** Characteristics of model substances.

Explosive	*H* [22,23]	$C_{sat}$, g/cm^3^ [24,25,26]	*D_a_*, 10^−6^ m^2^/s [24]	*K_sw_*, cm^3^/g [21,27]	*Q*, kJ/mol [22,28]
TNT	4.1 × 10^−6^	1.1 × 10^−11^	5.76	10.2	23
PETN	8.2 × 10^−8^	5.7 × 10^−13^	4.94	5.4	14
RDX	4.1 × 10^−9^	7.0 × 10^−14^	5.89	5.4	18

**Table 2:** Summary of experimental conditions and observed TNT vapor concentrations.

Experimental Condition	Sand Thickness	Moisture	Temperature	TNT Vapor Concentration/Response	Replicates
Dry untreated sand	1 cm	≤0.1%	room T	Not detected	6
Moistened untreated sand	1 cm	~11%	room T	~3 × 10^−13^ g cm^−3^	6
Dry washed sand	5 mm	≤0.1%	room T	~3 × 10^−14^ g cm^−3^	6
Highly moistened washed sand	4 cm	~40%	room T	Background level	6
Washed sand, decreasing temperature	1 cm	dry	29 → 19 °C	~3× background	6

## Data Availability

The original contributions presented in this study are included in the article. Further inquiries can be directed to the corresponding author.

## Abbreviations

The following abbreviations are used in this manuscript:

2,4-DNT	2,4-dinitrotoluene
GC	gas chromatography
GC–ECD	gas chromatography with electron capture detector
GC–MS	gas chromatography–mass spectrometry
IMS	ion mobility spectrometry
PETN	pentaerythritol tetranitrate
RDX	hexahydro-1,3,5-trinitro-1,3,5-triazine
TNT	2,4,6-trinitrotoluene

## Nomenclature

Symbol	Definition	Units
*C*	Vapor concentration in pore space	mol m^−3^
*C_a_*	Vapor concentration in the air phase	mol m^−3^
*C_w_*	Concentration in the aqueous phase	mol m^−3^
*C_sat_*	Saturated vapor concentration	mol m^−3^
*D_a_*	Diffusion coefficient in air	m^2^ s^−1^
*D_w_*	Diffusion coefficient in water	m^2^ s^−1^
*D_eff_*	Effective diffusion coefficient	m^2^ s^−1^
*D*	Delayed effective diffusion coefficient	m^2^ s^−1^
*R_f_*	Retardation factor	–
*H*	Henry’s law constant	–
*K_sa_*	Gas–solid partition coefficient	m^3^ kg^−1^
*K_sw_*	Water–solid partition coefficient	m^3^ kg^−1^
*k_d_*	First-order biodegradation rate constant	s^−1^
*L*	Burial depth	m
*t_st_*	Characteristic transport time	s
*z*	Distance from the explosive source	m
*t*	Time	s
ε*_a_*	Air-filled porosity	–
ε*_w_*	Water-filled porosity	–
ε*_T_*	Total porosity	–
ϕ	Volumetric soil moisture	–
ρ*_b_*	Bulk density	kg m^−3^
ρ*_s_*	Particle density	kg m^−3^
*Q*	Heat of sorption	kJ mol^−1^
*R*	Universal gas constant	J mol^−1^ K^−1^
*T*	Absolute temperature	K

## Appendix A

The TNT vapor concentration on the sand surface in all experiments was determined based on the amplitude of the TNT peak in the chromatogram. The peak amplitude was determined from six measurements. An example of a TNT vapor chromatogram from a 4.6 × 10^−14^ g/cm^3^ vapor concentration sensor at 20 °C is shown in Figure A1.

Figure A2 shows an example of a vapor chromatogram above a 1 cm layer of moistened, untreated sand.

## References

[1] Ding X.H., Feng S.J., Zheng Q.T., Peng C.H., Zhu Z.W., Yang C.B.X. (2022). Transient migration behavior of VOC vapor in layered unsaturated soils subjected to multiple time-dependent point pollution sources: Analytical study. Sci. Total Environ..

[2] Grossman S.L., Hutchinson K. (2003). Study of explosive residues found above buried landmines. Proceedings of the SPIE Detection and Remediation Technologies for Mines and Minelike Targets VIII, Orlando, FL, USA, 21–25 April 2003.

[3] Phelan J.M., Webb S.W. (1998). Simulation of the environmental fate and transport of chemical signatures from buried land mines. Proceedings of the SPIE Detection and Remediation Technologies for Mines and Minelike Targets III, Orlando, FL, USA, 13–17 April 1998.

[4] Phelan J.M., Barnett J.L., Romero J.V., Kerr D.R., Griffin F.A. (2003). Explosive chemical emissions from landmines. Proceedings of the SPIE Detection and Remediation Technologies for Mines and Minelike Targets VIII, Orlando, FL, USA, 21–25 April 2003.

[5] Amiel E. (2023). Enhancing genetically engineered Escherichia coli bioreporters for the detection of buried TNT-based landmines. bioRxiv.

[6] Lefferts M.J., Castell M.R. (2015). Vapour sensing of explosive materials. Anal. Methods.

[7] Yagur-Kroll S., Amiel E., Rosen R., Belkin S. (2015). Detection of 2,4-dinitrotoluene and 2,4,6-trinitrotoluene by an Escherichia coli bioreporter: Performance enhancement by directed evolution. Appl. Microbiol. Biotechnol..

[8] Kabessa Y., Eyal O., Bar-On O., Korouma V., Yagur-Kroll S., Belkin S., Agranat A.J. (2016). Standoff detection of explosives and buried landmines using fluorescent bacterial sensor cells. Biosens. Bioelectron..

[9] Prante M., Ude C., Große M., Raddatz L., Krings U., John G., Belkin S., Scheper T. (2018). A portable biosensor for 2,4-dinitrotoluene vapors. Sensors.

[10] Phelan J.M., Webb S.W., Gozdor M., Cal M., Barnett J.L. (2001). Effect of soil wetting and drying on DNT vapor flux: Laboratory data and T2TNT model comparisons. Proceedings of the SPIE Detection and Remediation Technologies for Mines and Minelike Targets VI, Orlando, FL, USA, 16–20 April 2001.

[11] Seigneur N., Mayer K.U., Steefel C.I. (2019). Reactive transport in evolving porous media. Rev. Miner. Geochem..

[12] Savatorova V., Talonov A. (2019). Mathematical modeling of gas transport in porous geological media with contrast of properties and irregular distribution of pores. ZAMM-J. Appl. Math. Mech..

[13] Larson S.L., Martin A.W., Escalon B.L., Thompson M. (2008). Dissolution, sorption, and kinetics involved in systems containing explosives, water, and soil. Environ. Sci. Technol..

[14] Pawłowski W., Karpińska M. (2021). The effect of soil moisture on the ability to detect TNT pairs from the sand layer in order to prevent environmental pollution and groundwater. Molecules.

[15] Albert M.R., Cragin J.H., Leggett D.C. (1999). Modeling explosive vapor transport through porous media. Proceedings of the SPIE Detection and Remediation Technologies for Mines and Minelike Targets IV, Orlando, FL, USA, 5–9 April 1999.

[16] Irrazábal M., Hernández-Rivera S.P., Briano J.G. (2009). Modeling of the transport of explosive related compounds in soil. Proceedings of the SPIE Detection and Sensing of Mines, Explosive Objects, and Obscured Targets XIV, Orlando, FL, USA, 13–17 April 2009.

[17] Ahmadi N., Heck K., Rolle M., Helmig R., Mosthaf K. (2021). On multicomponent gas diffusion and coupling concepts for porous media and free flow: A benchmark study. Comput. Geosci..

[18] Baqer Y., Chen X. (2022). A review on reactive transport model and porosity evolution in the porous media. Environ. Sci. Pollut. Res..

[19] Brust D., Hopf K., Fuhrmann J., Cheilytko A., Wullenkord M., Sattler C. (2025). Transport of heat and mass for reactive gas mixtures in porous media: Modeling and application. Chem. Eng. J..

[20] Kudryashova O.B., Sokolov S.D., Vorozhtsov A.B., Mikhailov Y.M., Gruznov V.M. (2024). Mathematical modeling in the problem of detecting explosive vapors from a source in soil. Izv. Vyss. Uchebnykh Zaved. Fiz..

[21] Ravikrishna R., Valsaraj K.T., Price C.B., Brannon J.M., Hayes C.A., Yost S.L. (2004). Vapor-phase transport of explosives from buried sources in soils. J. Air Waste Manag. Assoc..

[22] Phelan J.M., Barnett J.L., Parker D.R. (2002). Vapor Sensing Performance of the University of Missouri Rolla Explosive Vapor Sensor.

[23] Ariyarathna T., Vlahos P., Tobias C., Smith R. (2016). Sorption kinetics of TNT and RDX in anaerobic freshwater and marine sediments: Batch studies. Environ. Toxicol. Chem..

[24] Hikal W.M., Weeks B.L. (2014). Sublimation kinetics and diffusion coefficients of TNT, PETN, and RDX in air by thermogravimetry. Talanta.

[25] Schwarzenbach R.P., Gschwend P.M., Imboden D.M. (2016). Environmental Organic Chemistry.

[26] Ewing R.G., Waltman M.J., Atkinson D.A., Grate J.W., Hotchkiss P.J. (2013). The vapor pressures of explosives. TrAC Trends Anal. Chem..

[27] Dontsova K.M., Hayes C., Pennington J.C., Porter B. (2009). Sorption of high explosives to water-dispersible clay: Influence of organic carbon, aluminosilicate clay, and extractable iron. J. Environ. Qual..

[28] Zhuang L., Gui L., Gillham R.W. (2008). Degradation of pentaerythritol tetranitrate (PETN) by granular iron. Environ. Sci. Technol..

[29] Chiou C.T., Shoup T.D. (1985). Soil sorption of organic vapors and effects of humidity on sorptive mechanism and capacity. Environ. Sci. Technol..

[30] Petersen L.W., Moldrup P., El-Farhan Y.H., Jacobsen O.H., Yamaguchi T., Rolston D.E. (1995). The effect of moisture and soil texture on the adsorption of organic vapors. J. Environ. Qual..

[31] Smith J.A., Chiou C.T., Kammer J.A., Kile D.E. (1990). Effect of soil moisture on the sorption of trichloroethene vapor to vadose-zone soil at Picatinny Arsenal, New Jersey. Environ. Sci. Technol..

[32] Millington R.J., Quirk J.P. (1961). Permeability of porous solids. Trans. Faraday Soc..

[33] Goss K.U. (2005). Predicting the equilibrium partitioning of organic compounds using just one linear solvation energy relationship (LSER). Fluid Phase Equilib..

[34] Sander R. (2023). Compilation of Henry’s law constants (version 5.0.0) for water as solvent. Atmos. Chem. Phys..

[35] Bird R.B. (2002). Transport phenomena. Appl. Mech. Rev..

[36] (2014). Sand for Construction Works. Specifications.

[37] (1979). TNT for Industrial Explosives. Specifications.

[38] Gruznov V.M., Baldin M.N., Pryamov M.V., Maksimov E.M. (2017). Determination of explosive vapor concentrations with remote sampling in the control of objects. J. Anal. Chem..

